# From seed centres to tray cells: a geometry-aware machine vision framework for full-tray sowing quality assessment in maize plug trays

**DOI:** 10.3389/fpls.2026.1981493

**Published:** 2026-10-23

**Authors:** Jiangchen Zan, Jinfeng Peng, Ruijun Gao, Xiangyu Ma, Bowen Xue, Xiaoying Zhang

**Affiliations:** Faculty of Software Technologies, Shanxi Agricultural University, Jinzhong, Shanxi, China

**Keywords:** adaptive grid assignment, heatmap regression, machine vision, maize seedling tray, precision agriculture, seed-centre localisation, sowing quality assessment

## Abstract

Accurate sowing-quality assessment of maize seedling trays requires both reliable seed localisation and correct correspondence between detected seeds and physical tray cells. This study presents a semi-automated machine-vision framework combining heatmap-based seed-centre localisation, sliding-window inference, global non-maximum suppression (NMS), and four-corner-calibrated adaptive grid assignment. Each assessable cell was classified as empty, single-seeded, or multi-seeded according to the number of assigned seed centres. On source-image-independent subsets, the framework achieved an accuracy of 0.9821 and a macro F1-score of 0.9040 for 27 complete-tray images (3,456 cells), and 0.9798 and 0.9344, respectively, for 9 visible-cell images (1,140 assessable cells). Separately, the larger evaluation sets yielded accuracy/macro F1 values of 0.9783/0.9145 for 44 complete trays and 0.9782/0.9368 for 30 visible-cell images. These results support cell-level sowing-quality assessment under the evaluated acquisition conditions and within manually assessable regions. The source-image-independent subsets control direct source overlap but remain within the same acquisition domain; external generalisation across production sites, imaging devices, tray specifications, and acquisition conditions has not yet been established.

## 1 Introduction

Plug trays have become the fundamental production unit in greenhouse seedling cultivation because their standardized cell structures enable precise seed placement, uniform seedling management, and compatibility with automated nursery operations (Gao J. et al., 2022). Sowing uniformity directly influences seedling emergence and early growth consistency, making accurate evaluation of individual cell occupancy essential for refined nursery management (Li et al., 2025). However, manual inspection of large numbers of tray cells remains labour-intensive and vulnerable to subjective variation, limiting its application in large-scale seedling production (Yan et al., 2023). Therefore, automated approaches capable of identifying empty, single-seeded, and multi-seeded cells are increasingly required for precision plug-tray production.

Recent advances in computer vision and deep learning have significantly improved agricultural image analysis by enabling automated perception of complex biological targets and production environments (Kamilaris and Prenafeta-Boldú, 2018). Deep learning-based methods have been widely applied in crop monitoring, plant phenotyping, disease recognition, and agricultural object detection, demonstrating strong potential for intelligent agricultural systems (Yang and Xu, 2021; van Klompenburg et al., 2020). In controlled-environment agriculture, vision-based monitoring has further supported automated management by providing non-contact information acquisition and real-time analysis capabilities (Luo et al., 2024; Vasconez et al., 2019). However, most existing agricultural vision studies mainly focus on object-level detection or classification, whereas the transformation of detected targets into meaningful production-unit information remains insufficiently explored.

Plug-tray sowing assessment represents a different problem from conventional agricultural object detection. The objective is not only to locate individual seeds but also to determine the occupancy state of each physical tray cell. Previous studies have investigated missed-seeding detection, seed recognition, and seedling quality classification in plug trays (Gao L. et al., 2022; Du et al., 2022; Silwal et al., 2021). However, seed localisation alone cannot directly provide production decisions because detected seeds must be spatially associated with corresponding cells before empty, single-seeded, and multi-seeded states can be determined (Powers, 2011).

Full-tray image analysis introduces additional challenges due to the small size of individual seeds, complex backgrounds, and geometric variations during image acquisition. Small-object detection remains difficult in high-resolution agricultural images because target appearance can be easily affected by resolution reduction, background interference, and scale variation (Akyon et al., 2022). Meanwhile, practical greenhouse imaging commonly involves tray displacement, perspective distortion, incomplete boundaries, and illumination variation, which complicate the correspondence between image coordinates and physical tray structures (Tong et al., 2018).In addition, patch-based inference strategies may generate duplicate responses in overlapping regions, whereas fixed grid partitioning may produce incorrect cell assignments when tray geometry deviates from ideal rectangular assumptions (Koirala et al., 2019; Shorten and Khoshgoftaar, 2019; Sokolova and Lapalme, 2009). Therefore, a full-tray sowing assessment framework requires coordinated solutions for seed-centre localisation, prediction fusion, and geometry-aware cell assignment.

To address these challenges, this study proposes a geometry-aware, semi-automated framework that converts local seed-centre responses into cell-level sowing states. Its main contributions are: (1) heatmap-based seed-centre localisation with sliding-window inference for high-resolution tray images; (2) global duplicate suppression after coordinate restoration to prevent cross-window repeated counting; and (3) four-corner-calibrated adaptive grid assignment that converts retained centres into empty, single-seeded, and multi-seeded cell states. The framework is evaluated at patch, cell, and tray levels using controlled model comparison, module ablation, clustered uncertainty analysis, and source-overlap-controlled validation.

## 2 Materials and methods

### 2.1 Study site and image acquisition

Maize seedling tray images were collected from a real greenhouse seedling production system at the Northern China Forest and Fruit Seedling Breeding Base in Jinzhong, Shanxi Province, China. The experimental objects were standard plug trays with an 8 × 16 grid structure, containing 128 cultivation cells for maize seedling production.

Approximately 800 high-resolution images were acquired after sowing using an iPhone 16 Pro Max. Images were captured from a fixed position approximately 50–60 cm above the trays with a slightly inclined shooting angle. No additional control was imposed on greenhouse illumination, background conditions, or tray placement to preserve realistic production scenarios. The dataset therefore contains variations in illumination, substrate appearance, tray pose, perspective distortion, incomplete tray boundaries, and scenes involving multiple trays.

The collected images were used for patch generation, seed-centre annotation, and full-tray cell-level evaluation. The relationships among original images, generated patches, annotated samples, and evaluation subsets are summarized in **Table 1**. Source-image overlap between training patches and evaluation images was audited, and source-image-independent subsets were constructed for supplementary validation (Section 3.6).

**Table 1:** Raw data acquisition and source definition.

Data level	Quantity/scale	Filtering or annotation	Main use
Original greenhouse images	Approx. 800 images	Complete trays, incomplete tray boundaries, multiple trays in one frame, camera-pose variation, and complex backgrounds	Source of image patches and full-tray evaluation samples
Sliding-window patch pool	Approx. 70,000 patches	Local cell regions, substrate, seeds, and background-interference regions	Candidate source for subsequent filtering
Filtered patch candidates	Approx. 6,000 patches	Seed-containing, empty, complex negative, edge-seed, and difficult samples preferentially retained	Source for final annotation
Final annotated patch dataset	1,523 patch images and 2,681 seed centres	Visible seed centres manually annotated; split into training, validation, and testing sets	Training, validation, and testing of MSRA-HeatmapNet
Complete-tray evaluation samples	44 images and 5,632 cells	All cell states manually checked	Main complete-tray cell-level evaluation
Visible-cell evaluation samples	30 images and 3,808 valid visible cells	Only cells with visible = 1 and assessable sowing state evaluated	Evaluation within manually assessable visible regions

### 2.2 Dataset construction and annotation

#### 2.2.1 Patch-level seed-centre detection dataset

The patch-level dataset was constructed for training and evaluating the local seed-centre detector. Original full-tray images were cropped using a sliding-window strategy to preserve seed appearance, tray-cell structure, substrate texture, and local background information at sufficient spatial resolution.

Visible maize seeds were annotated using centre points rather than bounding boxes or segmentation masks, because subsequent cell-level classification relies on the number of seed centres assigned to each tray cell. Seed-containing patches were annotated with visible centres, whereas empty-cell and negative patches were assigned empty annotations.

To improve robustness under realistic greenhouse conditions, five representative patch categories were retained during sample screening: visible seed patches, empty-cell patches, complex negative patches with substrate interference, boundary seed patches, and difficult samples prone to detection errors (**Figure 1**). This strategy increased the diversity of local appearances and improved model adaptation to challenging image regions.

**Figure 1:**
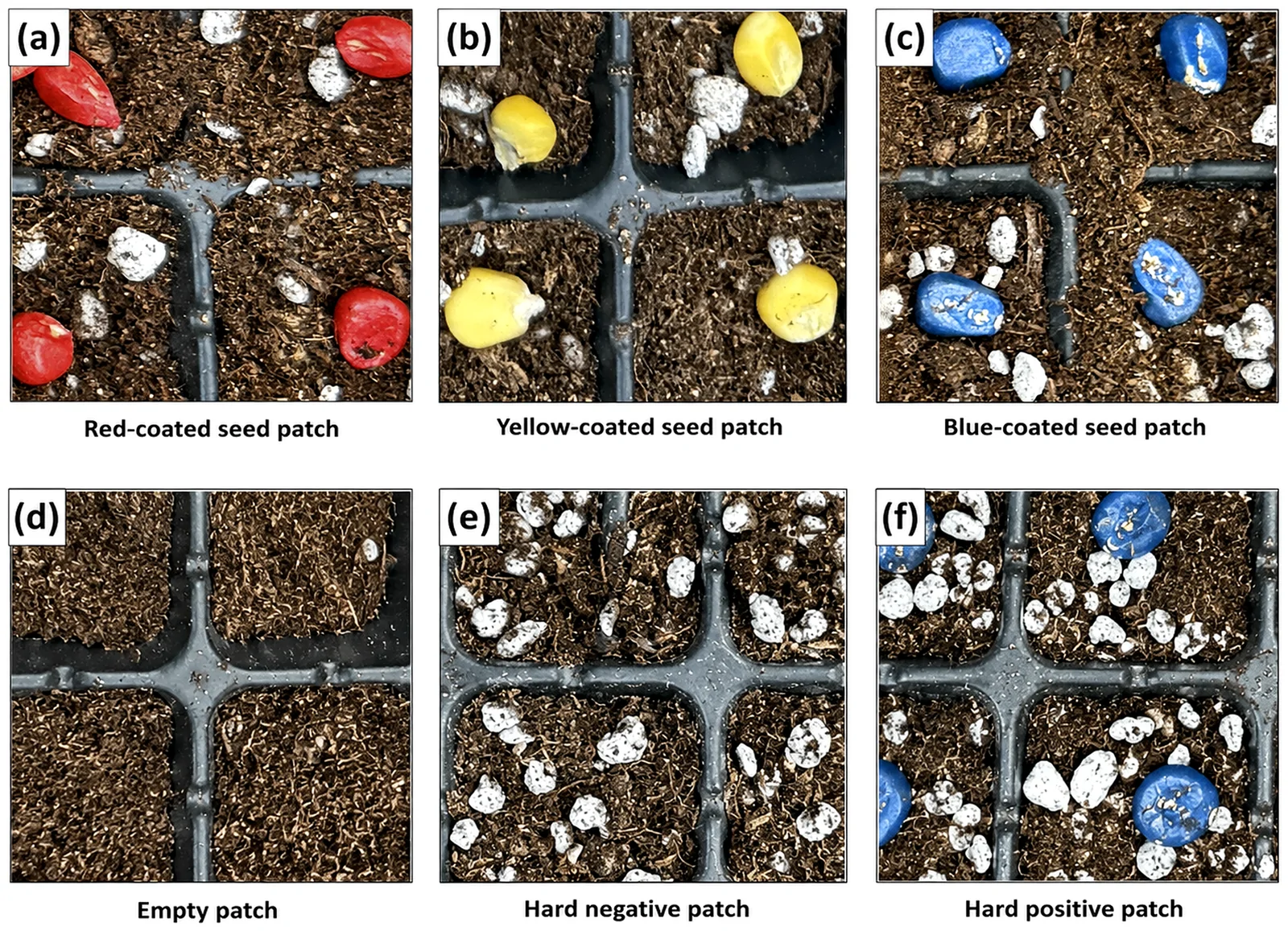
Examples of the dataset and complex image patches. **(a)** Red-coated seed patch; **(b)** yellow-coated seed patch; **(c)** blue-coated seed patch; **(d)** empty patch; **(e)** hard negative patch; **(f)** hard positive patch.

The final dataset_v3_labelled contained 1,523 manually verified patch images and 2,681 seed-centre annotations (**Table 2**). The retained development split was used for model optimisation, checkpoint selection, response-threshold selection, and patch-level development evaluation. Subsequent auditing of source-image metadata showed that patches derived from some parent images occurred in more than one development subset; therefore, this original patch split is not treated as source-image independent. Source independence was addressed separately through the controlled architecture experiment and the strict full-tray validation subsets described below.

**Table 2:** Dataset construction, filtering, annotation, and evaluation split.

Subset	Images	Seed-containing images	Blank images	Complex negative samples	Seed-centre points
Training	1,156	750	133	273	2,039
Validation	182	112	22	48	317
Testing	185	113	23	49	325
Total	1,523	975	178	370	2,681

To isolate the effect of network architecture, an additional controlled comparison was constructed from the same dataset_v3_labelled pool using a source-image-grouped partition. HeatmapNet and MSRA-HeatmapNet were trained with identical data partitions, augmentation, loss, optimiser, learning-rate schedule, random seed, early-stopping policy, and evaluation protocol. For each architecture, the response threshold was selected independently on the same validation set using the same threshold-sweep procedure and then applied once to the same test set. This controlled experiment was used only to assess architectural effects and did not replace the MSRA-HeatmapNet checkpoint used in the downstream full-tray experiments.

#### 2.2.2 Full-tray cell-level evaluation dataset

Because patch-level evaluation measures only local centre localisation, a separate full-tray dataset was used to evaluate the complete transformation from local predictions to tray-cell sowing states.

According to tray completeness and cell visibility, the evaluation dataset was divided into a complete-tray main evaluation set and a visible-cell evaluation set. The complete-tray set consisted of complete or near-complete single-tray images, with all 128 cells available for evaluation. A total of 44 tray images containing 5,632 cells were included, comprising 16 de-duplicated images from the original complete-tray samples (A_full_unique) and 28 complete target-tray images cropped from multi-tray scenes (A_target_cropped). Duplicate checking was performed on the original samples to remove repeated images and reduce potential evaluation bias.

The visible-cell evaluation set was designed to assess cell-state prediction in partially visible or geometrically non-ideal tray images while restricting quantitative analysis to manually assessable cells. Only cells with visible = 1 and reliable sowing-state annotations were included. The final quantitative subset contained 30 images and 3,808 of 3,840 cells. Twenty-five images had all 128 cells assessable, whereas five images contained 120–124 assessable cells because several edge cells could not be evaluated reliably. The median number of assessable cells per image was 128 (IQR: 128–128; range: 120–128), corresponding to a mean assessable-cell proportion of 99.17%. Four additional images with unreliable grid calibration or unclear boundaries were excluded from quantitative evaluation and retained for qualitative inspection. Accordingly, this dataset primarily evaluates performance within manually assessable regions under limited edge loss and non-ideal geometry rather than under severe boundary loss.

A visibility indicator was additionally assigned to the visible-cell subset. To further examine the influence of source-image overlap, stricter source-image-independent subsets were constructed by excluding evaluation samples derived from source images used for patch-level training. The composition of the full-tray evaluation datasets is summarized in **Table 3**.

**Table 3:** Full-tray cell-level evaluation datasets.

Evaluation set	Images	Cells	Evaluation protocol	Purpose
Complete-tray main set	44	5,632	All 128 cells per tray	Main experiment
Visible-cell evaluation set	30	3,808	Cells with visible = 1	Evaluation within manually assessable visible regions
Complete-tray independent subset	27	3,456	Samples with source IDs overlapping training images excluded	Data-independence validation
Visible-cell independent subset	9	1,140	Samples with source IDs overlapping training images excluded	Data-independence validation

### 2.3 Overall workflow

The proposed semi-automated framework converts local seed-centre detections into tray-cell sowing states through three sequential stages (**Figure 2**). First, sliding-window heatmap inference preserves local seed detail in high-resolution tray images. Second, predictions from overlapping windows are restored to the full-tray coordinate system and consolidated using global NMS. Third, an adaptive grid calibrated from four manually identified corner-cell centres assigns the retained seed centres to physical tray cells, from which empty, single-seeded, and multi-seeded states are derived.

**Figure 2:**
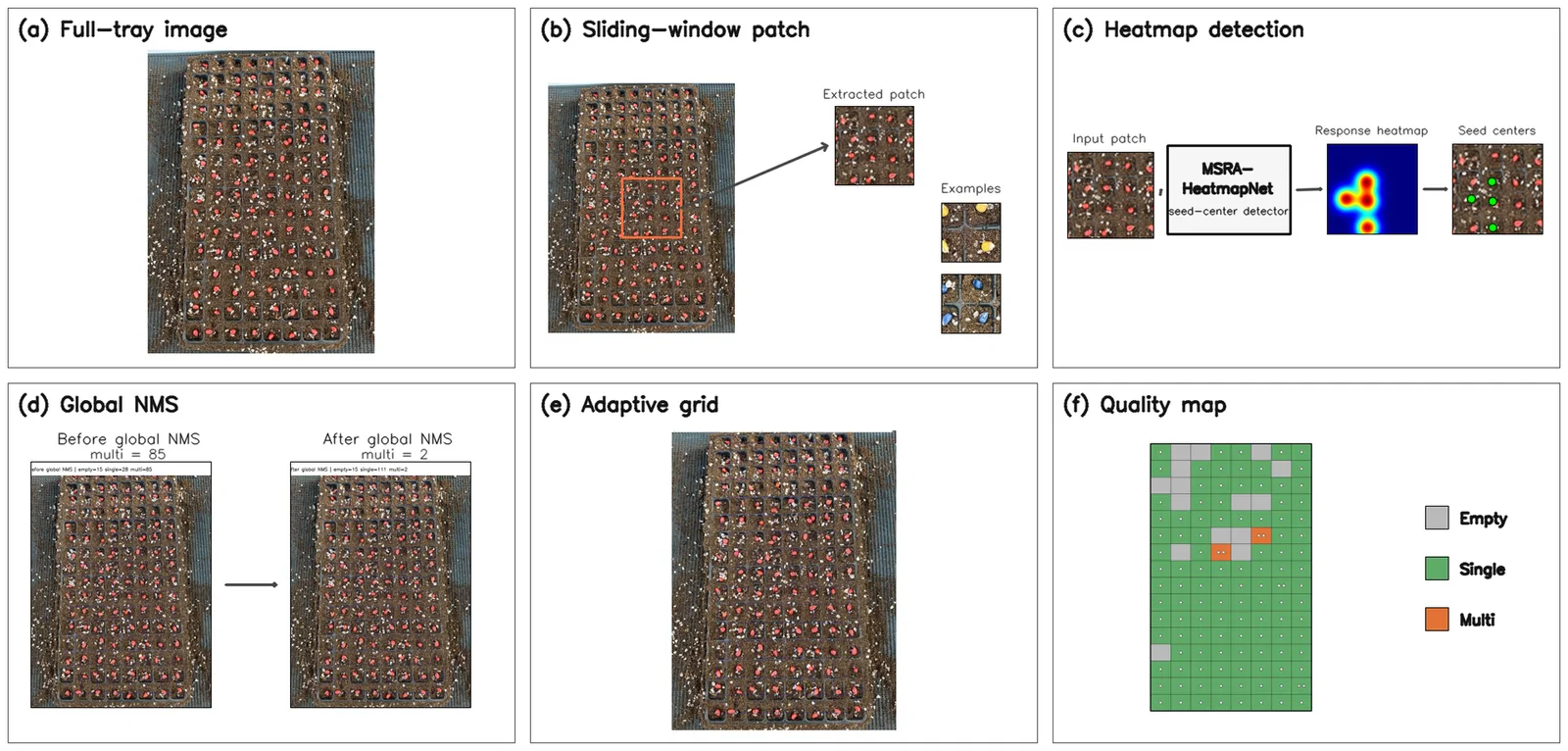
Semi-automated workflow for full-tray maize sowing-quality assessment. **(a)** Full-tray image; **(b)** sliding-window patches; **(c)** heatmap-based seed-centre detection; **(d)** global NMS; **(e)** adaptive grid generated from four manually calibrated corner-cell centres; and **(f)** cell-level sowing-state map.

Seed detection, duplicate suppression, coordinate transformation, cell assignment, and sowing-state classification are computationally automated after geometric calibration. Manual input is required for the four-corner calibration and, for the visible-cell evaluation set, determination of which cells are sufficiently visible and assessable for quantitative analysis. The mathematical formulations defining the main steps of this pipeline are given in Equations 1–49.

### 2.4 Seed-centre representation via heatmap regression

#### 2.4.1 Seed-centre heatmap representation

In this study, maize-seed detection is represented as a seed-centre localisation problem. For each input patch, manually annotated seed-centre coordinates are transformed into two-dimensional Gaussian heatmaps, which are used to supervise the model in learning the spatial distribution of seed locations. Compared with bounding-box detection or contour segmentation, the heatmap representation is more appropriate for this task because subsequent cell-level sowing-quality assessment relies on the number of seed centres assigned to each tray cell rather than complete seed boundaries or object contours.

Let W and H denote the width and height of an input patch, and let the output heatmap size be S × S. For the i-th visible seed in the patch, the annotated centre coordinate and its mapping to the heatmap coordinate system are defined as

(1) $$p_{i}=(x_{i},y_{i})$$

(2) $$x_{i}^{'}=\frac{x_{i}}{W-1}(S-1)$$

(3) yi'=yiH−1(S−1)

For any heatmap location q = u, v, the Gaussian response generated by the i-th seed centre is

(4) Hi(q)=exp[−(u−xi')2+(v−yi')22σ2]

where sigma is the Gaussian-kernel standard deviation in heatmap pixels. When a patch contains multiple seeds, the final supervised heatmap keeps the maximum seed response at each location:

(5) $$H(q)=\underset{i}{max}H_{i}(q)$$

For blank patches and complex negative patches without labelled seed centres, the supervised heatmap is defined as

(6) H(q)=0

At each heatmap pixel, the final response is obtained by retaining the maximum value among all seed-centred Gaussian maps, thereby preserving the strongest local response of individual seeds without introducing intensity accumulation. The resulting continuous heatmap targets are subsequently used for response thresholding and local peak extraction to identify individual seed centres.

#### 2.4.2 Heatmap-based seed-centre prediction

MSRA-HeatmapNet takes local image patches as input and generates single-channel seed-centre response maps. The heatmap output represents the spatial likelihood of seed centres and is used for subsequent centre extraction and coordinate transformation.

During training, the network learns to generate high responses around annotated seed centres while suppressing background responses from empty and complex regions. During inference, candidate centres are extracted from high-response regions using the validation-set-determined threshold and local-peak searching. Each detected centre is recorded with its position and response intensity for subsequent full-tray coordinate integration and cell-level assignment. The complete procedure from input patches to extracted seed centres is illustrated in **Figure 3**.

**Figure 3:**
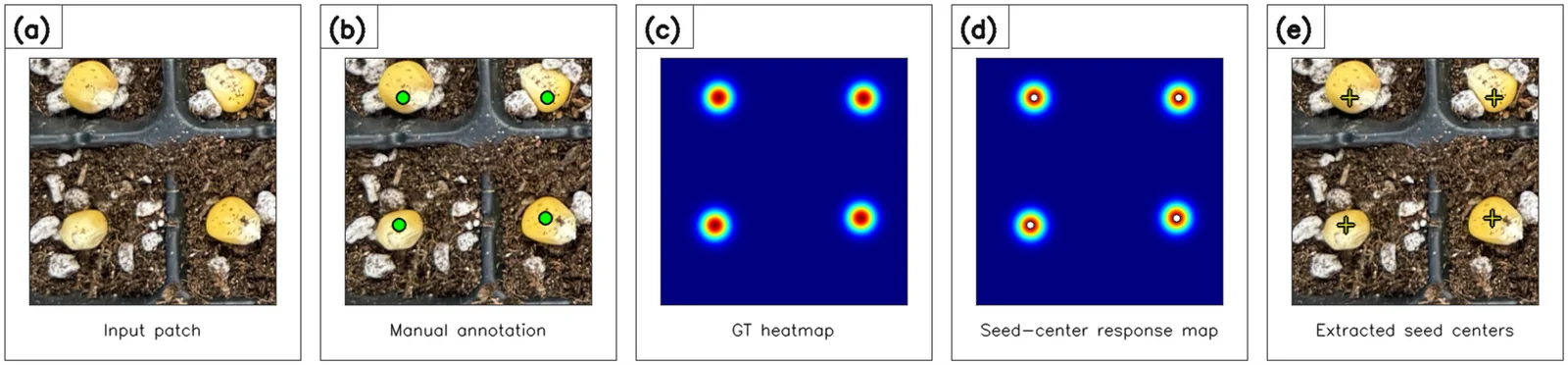
Illustration of MSRA-HeatmapNet seed-centre detection. **(a)** Input patch; **(b)** manually annotated seed centres; **(c)** ground-truth heatmap; **(d)** seed-centre response map; **(e)** extracted seed centres.

#### 2.4.3 Model optimization and seed-centre extraction

Training samples consisted of paired local image patches and their corresponding supervised heatmaps. The model parameters were optimized by minimizing the difference between predicted response maps and Gaussian target heatmaps, enabling concentrated responses around annotated seed centres while suppressing responses in unlabelled regions.

The original patch size was 512 × 512 pixels. To reduce computational cost while preserving local seed characteristics, input patches were resized to 256 × 256 pixels, and 128 × 128-pixel heatmaps were generated for supervision. The Gaussian target maps were constructed using a two-dimensional Gaussian kernel with a standard deviation of 2.5.

Data augmentation was applied only to the training set, including horizontal and vertical flipping, brightness and contrast variation, Gaussian blur, and Gaussian noise. No augmentation was applied to the validation or testing sets. The model was optimized using the AdamW optimizer with a combined loss consisting of weighted MSE and BCE heatmap terms. The weights for MSE, BCE, and BCE positive-class weighting were set to 1.0, 0.2, and 18.0, respectively. The checkpoint achieving the lowest validation loss was retained for subsequent experiments.

During inference, predicted response maps were processed using the response threshold determined from the validation set. Candidate seed centres were then extracted through local-peak searching with a minimum peak-distance constraint to avoid repeated detections within the same response region. Each extracted centre was recorded with its spatial coordinate and response intensity, and the resulting seed-centre candidates were transformed back to the full-tray coordinate system for subsequent global NMS-based duplicate removal. The training, extraction, and testing parameters are summarized in **Table 4**.

**Table 4:** Model training, full-tray inference, and grid-assignment parameters.

Parameter	Setting
Original patch size	512 × 512 pixels
Network input size	256 × 256 pixels
Output heatmap size	128 × 128 pixels
Gaussian kernel standard deviation	2.5
Optimiser	AdamW
Initial learning rate	0.001
Weight decay	0.0001
Learning-rate scheduler	ReduceLROnPlateau
Batch size	4
Loss function	Weighted MSE + BCE heatmap loss
Loss weights	MSE = 1.0; BCE = 0.2; BCE positive weight = 18.0
Response threshold	0.85
Minimum peak distance	6 heatmap pixels
Sliding-window size	512 × 512 pixels
Sliding-window stride	384 pixels
Window overlap	128 pixels (25%)
Internal edge margin	48 pixels
Global NMS distance	26 pixels
Grid-assignment tolerance	0.50 grid-cell units

The reported model training runs were executed in a CPU environment. The retained training logs for both HeatmapNet and MSRA-HeatmapNet recorded CPU execution, and no CUDA acceleration was used for the reported checkpoints. The computational environment comprised two Intel Xeon Platinum 8270 processors (2.70 GHz), 128 GB RAM, Windows 10.0.22631-SP0, Python 3.11.9, PyTorch 2.1.0+cpu, and OpenCV 4.13.0. The same CPU environment was used for the full-tray computational-efficiency benchmark reported in Section 3.7.

### 2.5 Full-tray seed-centre inference and coordinate integration

#### 2.5.1 Sliding-window inference on full-tray images

Full-tray images contain fine-grained seed details that may be lost when directly resized to the network input resolution. A 512 × 512-pixel sliding window with a stride of 384 pixels was therefore used, corresponding to a 128-pixel (25%) overlap between adjacent windows. The top-left coordinate of the k-th window in the original image is

(7) $$o_{k}=(a_{k},b_{k})$$

The corresponding local patch is cropped as

(8) $$I_{k}=I[b_{k}:b_{k}+L,\;a_{k}:a_{k}+L]$$

After Ik is processed by MSRA-HeatmapNet, the candidate seed-centre set extracted from that patch is

(9) $$C_{k}={\left(x_{k,j}^{p},y_{k,j}^{p},c_{k,j}\right)}_{j=1}^{n_{k}}$$

where (xk,jp, yk,jp) is the location of the j-th candidate seed centre in the coordinate system of the k-th patch, ck,j is the corresponding heatmap response, and nk is the number of candidate points extracted from that patch. Because downstream cell assignment is performed in the full-tray coordinate system, each patch-level candidate is mapped back to the original image as

(10) $$X_{k,j}=x_{k,j}^{p}+a_{k}$$

(11) $$Y_{k,j}=y_{k,j}^{p}+b_{k}$$

After candidate points from all patches are merged, the full-tray candidate seed-centre set is

(12) $$C^{raw}=\underset{k}{\cup }{\left\{(X_{k,j},Y_{k,j},c_{k,j})\right\}}_{j=1}^{n_{k}}$$

Because adjacent windows overlap, multiple responses may be generated for the same physical seed after merging patch-level predictions. To obtain a unique seed-centre set before cell-level counting, edge-margin filtering and global duplicate suppression were applied to remove unreliable boundary responses and duplicate detections.

#### 2.5.2 Global duplicate suppression of seed-centre predictions

Overlapping regions between adjacent sliding windows may generate multiple responses for the same physical seed after patch-level prediction merging. Directly using these responses for cell-level assessment would result in duplicated seed counts. Therefore, boundary filtering and global duplicate suppression were applied before seed-centre assignment.

For each candidate point in the local patch coordinate system, points located within a predefined edge margin were removed to reduce unreliable detections caused by truncated objects and insufficient contextual information. For windows located at the outer boundaries of the original full-tray image, candidates near the corresponding image boundaries were retained. In this study, the edge margin was set to 48 pixels, and the filtered candidate set was denoted as Cedge.

Global NMS was subsequently performed on Cedge after transforming all candidates into the full-tray coordinate system. Unlike local-peak extraction performed on individual heatmaps, global NMS considers all candidate points simultaneously to eliminate duplicate responses generated across overlapping windows. Candidates were sorted according to their response scores in descending order. For two candidate points pa= xa, ya and pb= xb, yb, the Euclidean distance was calculated as:

(13) $$D(p_{i},p_{j})=\sqrt{{\left(X_{i}-X_{j}\right)}^{2}+{\left(Y_{i}-Y_{j}\right)}^{2}}$$

Let R be the set of retained candidate points. After score sorting, a candidate point p is retained if

(14) $$\underset{p_{j}\in C_{keep}}{min}D(p_{i},p_{j})\geq d_{nms}$$

where dnms is the de-duplication distance threshold in the full-tray coordinate system. In this study, dnms was set to 26 pixels. If the distance between the current candidate and any retained point is smaller than this threshold, the current candidate is treated as a repeated response and is suppressed.

(15) $$C^{nms}={\left\{(X_{m},Y_{m},c_{m})\right\}}_{m=1}^{M}$$

where M is the number of retained seed centres after global NMS. The set Cnms is then passed to the adaptive grid assignment module to map seed centres to corresponding tray cells. **Figure 4** illustrates the effect of global NMS in removing duplicate candidate responses generated across overlapping patches in the full-tray coordinate system.

**Figure 4:**
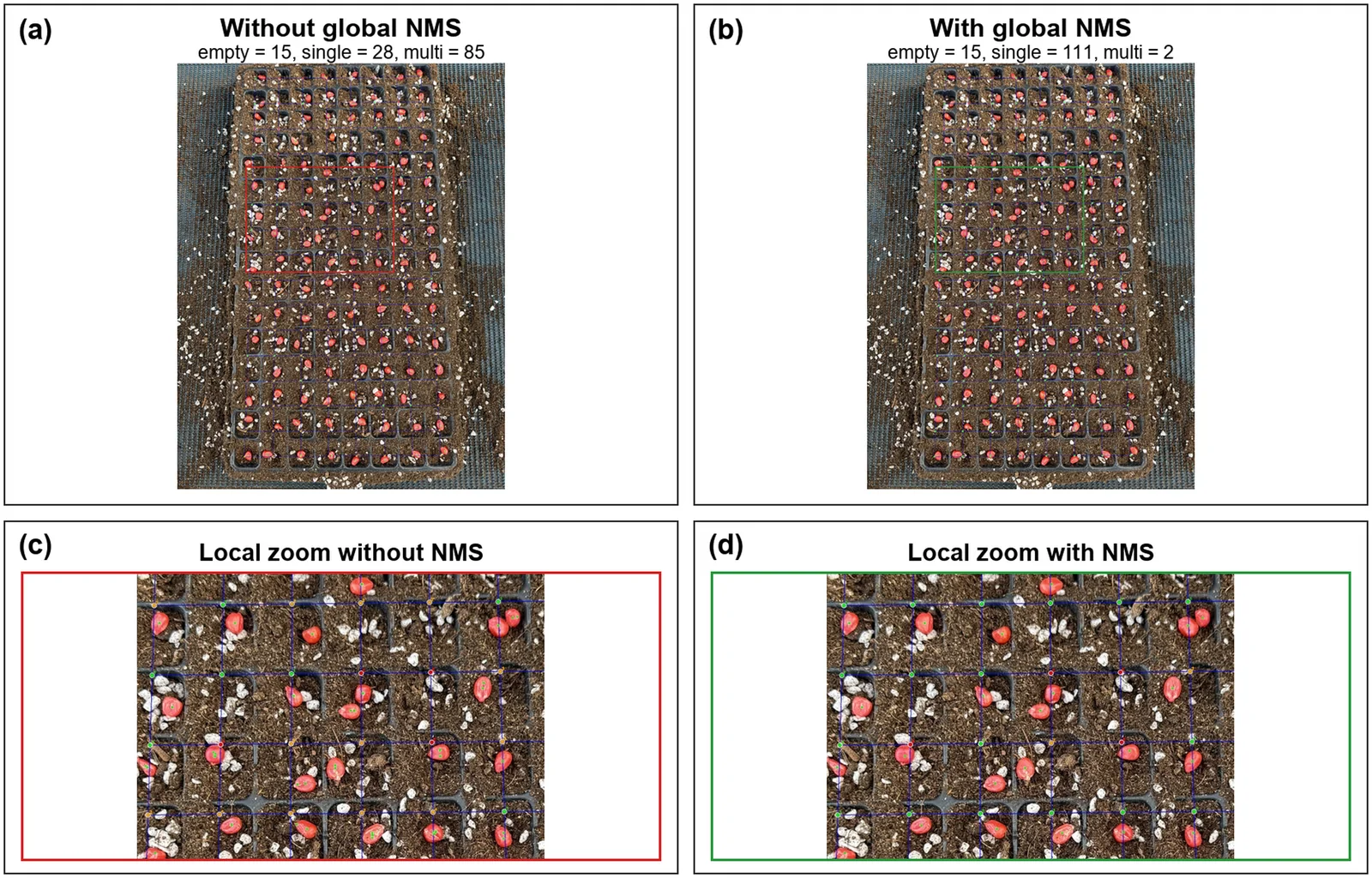
Effect of global NMS on duplicate seed-centre removal after sliding-window inference. **(a, c)** Without global NMS; **(b, d)** with global NMS in the full-tray coordinate system.

### 2.6 Geometry-aware seed-centre to tray-cell assignment

#### 2.6.1 Limitation of fixed-grid assignment

After global NMS, the retained seed centres represent reliable seed locations in the full-tray coordinate system. However, these point predictions still require a spatial assignment strategy to determine their corresponding tray cells for cell-level sowing-quality assessment.

Although plug trays follow a regular grid structure, their appearance in greenhouse images is affected by perspective distortion, camera pose variation, tray displacement, and incomplete boundaries. A fixed rectangular grid assumes uniform cell distribution and ignores such geometric variations, which may cause misalignment between predefined grid boundaries and actual cell locations. Consequently, correctly detected seed centres, particularly those near edges and corners, may be assigned to incorrect cells and affect the final sowing-state classification.

Therefore, this study employs a four-corner-calibrated adaptive grid to establish the correspondence between seed centres and tray cells under different tray poses. A fixed rectangular grid was retained only as a baseline in the ablation experiment to evaluate the contribution of adaptive grid assignment.

#### 2.6.2 Adaptive grid construction

For each tray image, the centres of the four corner cells were manually identified in the fixed order top-left, top-right, bottom-right, and bottom-left using a custom Python/OpenCV click-based interface. The resulting grid overlay was visually reviewed before seed-to-cell assignment. Calibration was performed by one operator and subsequently checked by a second person for quality control. The four corner-cell centres were recorded in the original full-tray image coordinate system as follows:

(16) $$P_{tl}=(x_{tl},y_{tl})$$

(17) $$P_{tr}=(x_{tr},y_{tr})$$

(18) $$P_{br}=(x_{br},y_{br})$$

(19) $$P_{bl}=(x_{bl},y_{bl})$$

All four corner points were defined in the original full-tray image coordinate system. Based on the relative lengths of the upper and left tray boundaries, the tray orientation was determined to select the corresponding grid arrangement. Therefore, the adaptive grid maintained the original 128-cell tray structure, represented as either Nr × Nc = 16 × 8 or Nr × Nc = 8 × 16, depending on the orientation in the image.

The adaptive grid construction defines a geometric transformation from the original image coordinate system to a normalized tray-grid coordinate system. The four target grid points corresponding to the calibrated tray corners are defined as follows:

(20) $$Q_{tl}=(0,0)$$

(21) $$Q_{tr}=(C-1,0)$$

(22) $$Q_{br}=(C-1,R-1)$$

(23) $$Q_{bl}=(0,R-1)$$

(24) H=Homography(P,Q)

where H denotes the homography matrix that transforms points from the original image coordinate system to the normalized grid coordinate system. For any de-duplicated seed-centre point pm = (Xm, Ym), its corresponding homogeneous grid coordinate is obtained as:

(25) $${\left[u{'}_{m},v{'}_{m},w{'}_{m}\right]}^{T}=H{\left[X_{m},Y_{m},1\right]}^{T}$$

After normalisation, um and vm are the continuous grid coordinates of the seed centre, where um and vm denote the continuous column and row directions, respectively.

#### 2.6.3 Seed-centre-to-cell assignment

After obtaining continuous grid coordinates, each seed centre was assigned to the corresponding tray cell by converting continuous coordinates into discrete row and column indices. The 0-based column and row indices were calculated as:

(26) $${\hat{c}}_{m}=round(u_{m})$$

(27) $${\hat{r}}_{m}=round(v_{m})$$

To avoid assigning points near grid boundaries or outside the valid tray region to incorrect cells, the distances between each seed centre and its assigned grid centre were further calculated as:

(28) $$\Delta u_{m}=|u_{m}-{\hat{c}}_{m}|$$

(29) $$\Delta v_{m}=|v_{m}-{\hat{r}}_{m}|$$

A seed centre was accepted only when its rounded row and column indices were within the valid tray range and the absolute deviations from the nearest grid centre in both directions were ≤0.50 grid-cell units. A direct implementation check on 28 tray images (2,839 fused seed points; 3,584 cells) showed that changing the previous non-binding value of 0.85 to 0.50 produced no change in point assignment, assigned cell index, or cell-level empty/single/multi classification:

(30) $$0\leq r_{m}<R$$

(31) $$0\leq {\hat{c}}_{m}<C$$

(32) $$\Delta v_{m}\leq \alpha$$

For each tray cell, the seed count is determined by the number of de-duplicated seed centres assigned to that cell. The seed count of the cell at row r and column c is defined as:

(33) $$N_{r,c}=\sum_{m=1}^{M}1({\hat{r}}_{m}=r,{\hat{c}}_{m}=c,A_{m}=1)$$

where Am indicates whether the m-th seed centre is successfully assigned, rm and cm are its assigned row and column indices, and 1(.) is the indicator function. Therefore, the de-duplicated seed-centre set in the full-tray coordinate system is converted into cell-level seed counts consistent with the tray structure. **Figure 5** illustrates the adaptive-grid-based seed-centre-to-cell assignment process.

**Figure 5:**
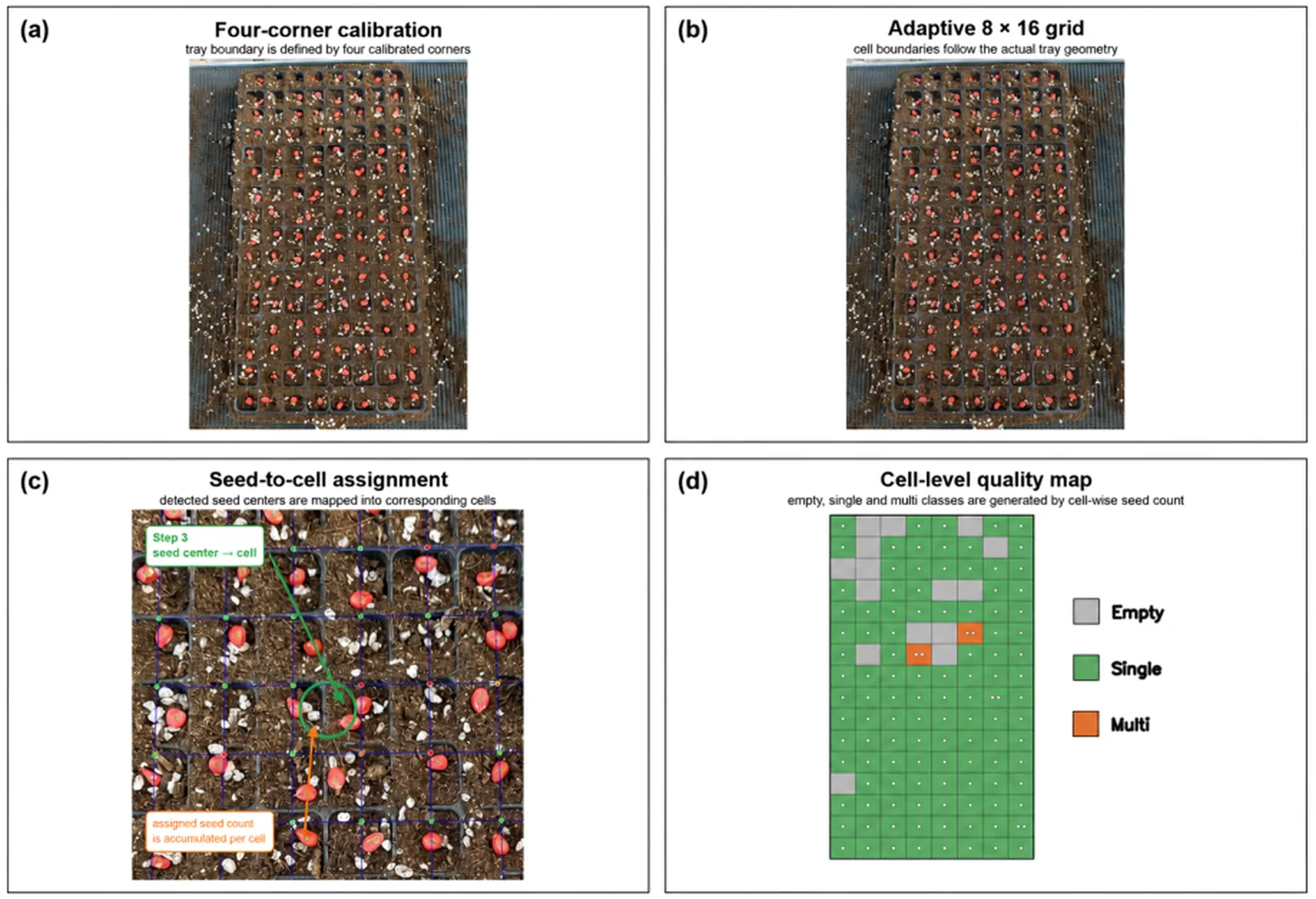
Seed-centre-to-cell assignment using a four-corner-calibrated adaptive grid. **(a)** Manual corner-cell-centre calibration; **(b)** adaptive grid construction; **(c)** seed-centre assignment to tray cells; and **(d)** resulting cell-level sowing-state map. Cells containing zero, one, or more than one assigned seed centre were classified as empty, single-seeded, and multi-seeded, respectively.

### 2.7 Cell-level sowing-state classification

After adaptive grid assignment, each tray cell is associated with a seed-centre count derived from the assigned de-duplicated seed centres. Let nr,c denote the seed count in the cell at row r and column c. The sowing state Sr,c is defined as

(34) $$S_{r,c}=\{\begin{array}{ll} empty, & N_{r,c}=0\\ single, & N_{r,c}=1\\ multi, & N_{r,c}\geq 2 \end{array}$$

Here, empty represents a cell without detected seed centres, single represents a cell containing one seed centre, and multi represents a cell containing multiple seed centres. This classification scheme reflects practical sowing conditions, where empty cells indicate missing occupancy, single cells represent the desired sowing state, and multi cells indicate potential over-seeding and competition among seedlings.

For the complete-tray main evaluation set, all tray cells were included in quantitative evaluation. For the visible-cell evaluation set, metrics were calculated only for cells previously designated as manually assessable in Section 2.2.2. The valid evaluated cell set is denoted as Ω.

(35) $$\Omega =\{(r,c)1\leq r\leq R,\;1\leq c\leq C,\;visible_{r,c}=1\}$$

The resulting cell-state map provides cell-level sowing states and supports tray-level sowing-quality assessment.

### 2.8 Evaluation metrics

Three levels of evaluation metrics were adopted to assess the proposed framework: patch-level seed-centre localisation, cell-level sowing-state classification, and tray-level sowing-quality errors. Precision, recall, F1-score, and accuracy were calculated following standard machine-learning evaluation protocols (Chen and Zhang, 2024). Macro F1 was reported to provide equal importance to the three sowing states, whereas weighted F1 was additionally calculated according to the observed class distribution.

#### 2.8.1 Patch-level seed-centre detection metrics

For patch-level seed-centre evaluation, the response threshold was first optimized on the validation set by scanning candidate thresholds and selecting the value achieving the highest validation F1-score. The selected threshold was then applied to the independent testing set.

A one-to-one matching strategy was used to associate predicted seed centres with manually annotated centres. Euclidean distances between all predicted and annotated points were calculated, and candidate pairs with distances ≤18 pixels were retained. Matching was subsequently performed in ascending order of distance, and matched points were excluded from further matching.

Let TP denote the number of correctly matched seed centres, FP the number of unmatched predicted centres, and FN the number of unmatched annotated centres. Precision, recall, and F1-score were calculated as:

(36) $$Precision=\frac{TP}{TP+FP}$$

(37) $$Recall=\frac{TP}{TP+FN}$$

(38) $$F1=\frac{2\times Precision\times Recall}{Precision+Recall}$$

If the denominator in a metric calculation is zero, the corresponding metric value was set to zero. In addition to standard detection metrics, the mean localisation error of matched seed centres and the counting error of each local image were recorded to further characterize localisation accuracy and local counting performance.

#### 2.8.2 Cell-level sowing-state classification metrics

Cell-level evaluation was performed using manually verified cell states as ground truth and predicted cell states as classification outputs. For the complete-tray main evaluation set, all tray cells were included in evaluation. For the visible-cell evaluation set, only cells with visible=1 and valid manual labels were considered. Let Ω denote the set of valid evaluated cells, and let K denote the set of sowing-state classes:

(39) K={empty,single,multi}

For each class k∈K, true positives, false positives, and false negatives were obtained from the confusion matrix. Class-level precision, recall, and F1-score were calculated as:

(40) $$Precision_{k}=\frac{TP_{k}}{TP_{k}+FP_{k}}$$

(41) $$Recall_{k}=\frac{TP_{k}}{TP_{k}+FN_{k}}$$

(42) $$F1_{k}=\frac{2\times Precision_{k}\times Recall_{k}}{Precision_{k}+Recall_{k}}$$

Overall accuracy was calculated as the proportion of correctly classified cells among all valid evaluated cells:

(43) $$Accuracy=\frac{1}{\left|\Omega \right|}\sum_{(r,c)\in \Omega }1(S_{r,c}^{pred}=S_{r,c}^{gt})$$

where Sr,cpred and Sr,cgt denote the predicted and manually verified ground-truth states of the cell at row r and column c, respectively. Macro F1 reflects the average performance across the three sowing states, whereas weighted F1 accounts for the observed class distribution.

(44) $$Macro\;F1=\frac{1}{\left|K\right|}\sum_{k\in K}F1_{k}$$

Weighted F1 was calculated by weighting each class-level F1-score according to the number of ground-truth cells in that class:

(45) $$Weighted\;F1=\frac{\sum_{k\in K}n_{k}F1_{k}}{\sum_{k\in K}n_{k}}$$

where nk represents the number of ground-truth cells belonging to class k. Macro F1 reflects the average performance across the three sowing states, whereas weighted F1 accounts for the observed class distribution.

#### 2.8.3 Tray-level sowing-quality rate errors

In addition to cell-level classification metrics, tray-level rate errors were calculated to evaluate whether the proposed framework could accurately estimate the distribution of sowing states within each tray. For the t−th tray image, let Ωt denote its valid evaluated cell set. For class k, the manually verified rate and predicted rate are defined as:

(46) $$R_{t,k}^{gt}=\frac{1}{\left|\Omega_{t}\right|}\sum_{(r,c)\in \Omega_{t}}1(S_{r,c}^{gt}=k)$$

(47) $$R_{t,k}^{pred}=\frac{1}{\left|\Omega_{t}\right|}\sum_{(r,c)\in \Omega_{t}}1(S_{r,c}^{pred}=k)$$

The absolute error of the sowing-state rate is calculated as:

(48) $$E_{t,k}=|R_{t,k}^{pred}-R_{t,k}^{gt}|$$

The mean absolute error across all evaluated tray images is calculated as:

(49) $$MAE_{k}=\frac{1}{T}\sum_{t=1}^{T}E_{t,k}\;\;$$

where T denotes the number of evaluated tray images. The empty-rate, single-rate, and multi-rate errors were reported to quantify the deviation between predicted and manually verified sowing-state distributions.

#### 2.8.4 Statistical uncertainty and paired comparisons

Because multiple cell-level observations originated from the same tray image, cells within a tray were treated as clustered observations rather than independent experimental units. Uncertainty in complete-tray performance was therefore quantified using a tray-level clustered bootstrap. For each bootstrap replicate, 44 tray images were sampled with replacement from the complete-tray evaluation set, and all cells belonging to each selected tray were retained together. Accuracy, macro F1-score, and class-specific F1-scores were recalculated from the pooled cells of the resampled trays. The 2.5th and 97.5th percentiles of 20,000 bootstrap replicates were used to construct 95% confidence intervals. For the ablation experiments, paired tray-level bootstrap resampling was used because the same tray images and cells were evaluated under both pipeline configurations. Within each replicate, an identical set of resampled trays was used for the two configurations, and the differences in accuracy and macro F1-score were calculated. Confidence intervals for the paired differences were obtained from the corresponding 2.5th and 97.5th percentiles. Tray-to-tray variability of the complete framework was additionally summarized using the median, interquartile range, and range of per-tray accuracy.

## 3 Results

### 3.1 Controlled patch-level architectural comparison

A separate controlled comparison was performed to isolate architectural effects. Both HeatmapNet and MSRA-HeatmapNet were trained on the same source-image-grouped dataset partition under identical optimisation and augmentation settings. Each model selected its response threshold on the same validation set and was evaluated once on the same 233-image test set containing 403 annotated seed centres (**Table 5**).

**Table 5:** Controlled architectural comparison on the common source-image-grouped patch test set.

Model	Selected threshold	TP/FP/FN	Precision	Recall	F1-score	Mean localisation error (px)	Mean absolute count error
HeatmapNet	0.45	366/83/37	0.8151	0.9082	0.8592	7.451	0.3262
MSRA-HeatmapNet	0.80	358/78/45	0.8211	0.8883	0.8534	7.518	0.2876

The two architectures showed comparable overall performance. MSRA-HeatmapNet achieved slightly higher precision (0.8211 vs 0.8151) and lower mean absolute count error (0.2876 vs 0.3262), whereas HeatmapNet achieved higher recall (0.9082 vs 0.8883) and F1-score (0.8592 vs 0.8534). Mean localisation error was similar (7.451 vs 7.518 pixels). Thus, the controlled experiment did not show a clear overall performance advantage for MSRA-HeatmapNet, and the improvement observed in the earlier development-stage comparison cannot be attributed solely to architecture.

### 3.2 Cell-level sowing-quality evaluation on complete trays

The complete-tray main evaluation set contained 44 complete or near-complete tray images and 5,632 manually checked cells. **Figure 6a** shows a representative prediction and the corresponding cell-level quality map.

**Figure 6:**
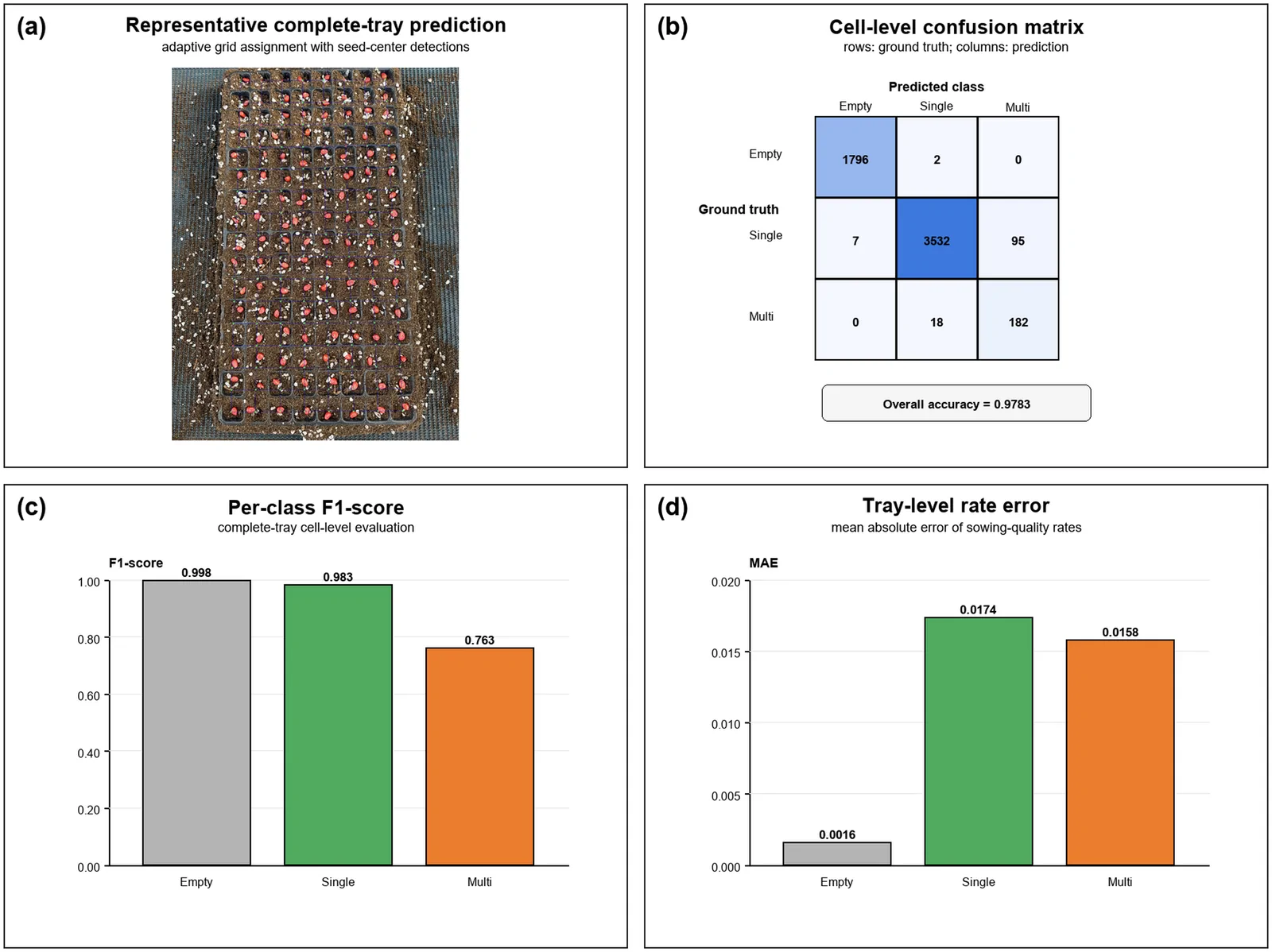
Cell- and tray-level sowing-quality evaluation on the complete-tray dataset. **(a)** Representative complete-tray prediction with adaptive grid assignment; **(b)** cell-level confusion matrix; **(c)** per-class F1-score; **(d)** tray-level sowing-quality rate error.

Across the 5,632 cells, the framework achieved an overall accuracy of 0.9783 and a macro F1-score of 0.9145. Tray-level clustered bootstrap analysis (20,000 replicates) yielded 95% confidence intervals of 0.9721–0.9842 for accuracy and 0.8912–0.9347 for macro F1-score (**Table 6**). The class-specific F1-scores were 0.9975 for empty cells, 0.9830 for single-seeded cells, and 0.7631 for multi-seeded cells, with corresponding clustered 95% confidence intervals of 0.9950–0.9992, 0.9790–0.9870, and 0.6950–0.8211, respectively. The framework correctly classified 1,796 of 1,798 empty cells, 3,532 of 3,634 single-seeded cells, and 182 of 200 multi-seeded cells. Per-tray accuracy had a median of 0.9844, an interquartile range of 0.9688–0.9922, and a range of 0.9297–1.0000.

**Table 6:** Tray-clustered bootstrap uncertainty for the complete-tray evaluation set.

Metric	Estimate	95% confidence interval
Overall accuracy	0.9783	0.9721–0.9842
Macro F1	0.9145	0.8912–0.9347
F1-empty	0.9975	0.9950–0.9992
F1-single	0.9830	0.9790–0.9870
F1-multi	0.7631	0.6950–0.8211

The mean absolute errors of the empty, qualified, and multi-seeded rates were 0.0016, 0.0174, and 0.0158, respectively (**Figure 6d**), where the qualified rate is the single-seeded rate. Thus, residual single–multi confusion had limited effect on aggregate rate estimation within the evaluated data. These rates are relevant to production monitoring, although their operational benefit for planter adjustment or re-seeding was not tested.

### 3.3 Evaluation under partial tray visibility

The partial-visibility analysis considered images with incomplete boundaries, view-angle deviations, or locally unassessable edge regions. Quantitative evaluation was restricted to cells manually confirmed as visible and assessable; cells with ambiguous boundaries or unreliable sowing-state labels were excluded rather than forced into the evaluation.

The visible-cell evaluation set contained 30 images and 3,808 assessable cells. Twenty-five images had all 128 cells assessable, while five contained 120–124 assessable cells. The median number of assessable cells per image was 128 (IQR: 128–128; range: 120–128), and the mean assessable-cell proportion was 99.17%. Within these manually assessable regions, the framework achieved an overall accuracy of 0.9782, a macro F1-score of 0.9368, and a weighted F1-score of 0.9793. The class-specific F1-scores were 0.9967 for empty cells, 0.9845 for single-seeded cells, and 0.8293 for multi-seeded cells (**Table 7**).

**Table 7:** Cell-level sowing-quality evaluation results on the visible-cell evaluation set.

Evaluation set	Images	Valid cells	Overall accuracy	Macro F1	Weighted F1	F1-empty	F1-single	F1-multi
Visible-cell robustness set	30	3,808	0.9782	0.9368	0.9793	0.9967	0.9845	0.8293

The mean absolute errors of the empty-cell, qualified-cell, and multi-seeded rates were 0.0016, 0.0165, and 0.0149, respectively. These results support stable sowing-state estimation within predominantly visible and manually assessable tray regions. They should not be interpreted as quantitative validation under severe boundary loss, because such cases were excluded when reliable calibration or cell-level ground truth could not be established.

### 3.4 Ablation analysis of global duplicate suppression

To verify the necessity of global duplicate suppression in the proposed framework, an ablation experiment was conducted by removing the global NMS module while keeping the seed-centre detector and adaptive grid assignment unchanged. The experiment was performed on the complete-tray evaluation set to assess the influence of cross-window duplicate predictions on cell-level sowing-quality assessment.

Without global NMS, 8,089 candidate points were retained, resulting in substantial degradation of cell-level classification performance (**Table 8**). Overall accuracy and macro F1 decreased to 0.5661 and 0.5435, respectively, with 2,444 incorrectly classified cells. The decrease was mainly observed in occupied-cell categories, where duplicate seed-centre responses caused over-counting and increased single–multi confusion.

**Table 8:** Ablation analysis of global duplicate suppression on full-tray cell-level evaluation.

Setting	Candidate points	Overall accuracy	Macro F1	Weighted F1	F1-empty	F1-single	F1-multi	Error cells
Without global NMS	8,089	0.5661	0.5435	0.6427	0.9975	0.4949	0.1380	2,444
With global NMS	4,226	0.9783	0.9145	0.9798	0.9975	0.9830	0.7631	122

After reintroducing global NMS, 3,863 redundant candidates (47.76%) were removed, reducing the candidate number to 4,226. The framework recovered an accuracy of 0.9783 and a macro F1-score of 0.9145, while erroneous cells decreased to 122. Paired tray-level bootstrap analysis showed increases of 0.4123 in accuracy (95% CI: 0.3587–0.4645) and 0.3711 in macro F1-score (95% CI: 0.3514–0.3901; **Table 9**). At the tray level, qualified-rate and multi-seeded-rate MAE decreased from 0.4316 and 0.4308 without NMS to 0.0174 and 0.0158 with NMS, respectively.

**Table 9:** Paired tray-level bootstrap effects of downstream modules on the complete-tray evaluation set.

Comparison	Metric	Difference	95% confidence interval
With NMS − without NMS	Accuracy	+0.4123	0.3587–0.4645
With NMS − without NMS	Macro F1	+0.3711	0.3514–0.3901
Adaptive grid − fixed grid	Accuracy	+0.2422	0.2195–0.2638
Adaptive grid − fixed grid	Macro F1	+0.3222	0.2955–0.3506

### 3.5 Ablation analysis of adaptive grid assignment for cell-level mapping

To evaluate the contribution of adaptive grid assignment to accurate seed-to-cell mapping, fixed rectangular grid assignment and adaptive grid assignment were compared on the complete-tray set using identical seed centres retained after global NMS.

The fixed grid achieved an overall accuracy of 0.7362, a macro F1-score of 0.5924, and 1,486 erroneous cells (**Table 10**). The multi-seeded class showed the largest degradation, with an F1-score of 0.2392, indicating that geometric misalignment mainly affected occupancy states determined by the number of assigned seed centres within individual cells.

**Table 10:** Ablation analysis of adaptive grid assignment for seed-to-cell mapping.

Setting	Overall accuracy	Macro F1	F1-single	F1-multi	MAE-qualified rate	MAE-multi rate	Error cells
Fixed rectangular grid	0.7362	0.5924	0.7789	0.2392	0.1355	0.0618	1,486
Adaptive grid	0.9783	0.9145	0.9830	0.7631	0.0174	0.0158	122

With adaptive grid assignment, overall accuracy increased to 0.9783 and macro F1-score to 0.9145, while erroneous cells decreased to 122. The F1-score of the multi-seeded class improved from 0.2392 to 0.7631. Paired tray-level bootstrap analysis showed increases of 0.2422 in accuracy (95% CI: 0.2195–0.2638) and 0.3222 in macro F1-score (95% CI: 0.2955–0.3506) relative to the fixed-grid configuration (**Table 9**). These results support the role of geometry-aware mapping in reducing seed-to-cell assignment errors under tray pose and perspective variation.

At the tray level, adaptive grid assignment also improved quality-rate estimation. The MAE values of empty-, qualified-, and multi-seeded rates decreased from 0.0740, 0.1355, and 0.0618 with the fixed grid to 0.0016, 0.0174, and 0.0158 with the adaptive grid, respectively.

### 3.6 Validation on source-image-independent subsets

To further examine the influence of source-image overlap, source-image-independent subsets were constructed by excluding evaluation samples whose source identifiers overlapped with training patch images. The evaluation procedure and metrics remained identical to those used in the main experiments, and the analysis was performed as an additional validation rather than a replacement of the original evaluation sets.

On the source-image-independent complete-tray subset, comprising 27 images and 3,456 cells, accuracy and macro F1 were 0.9821 and 0.9040, respectively. On the independent visible-cell subset, comprising 9 images and 1,140 assessable cells, the corresponding values were 0.9798 and 0.9344 (**Table 11**). The empty-, qualified-, and multi-seeded rate MAEs were 0.0014, 0.0162, and 0.0148 for the complete-tray subset and 0.0009, 0.0130, and 0.0122 for the visible-cell subset. In the 9-image visible-cell subset, 1,140 of 1,152 cells were assessable, with per-image assessable proportions ranging from 93.75% to 100%. These results are reported separately from the larger evaluation sets because they address direct source-image overlap. They do not establish external generalisation or performance under severe partial visibility, and the small visible-cell subset warrants cautious interpretation.

**Table 11:** Cell- and tray-level evaluation results on strict source-image-independent subsets.

Evaluation set	Images	Valid cells	Overall accuracy	Macro F1	Weighted F1	Class-wise F1 (empty/single/multi)	Rate MAE (empty/qualified/multi)
Complete-tray independent subset	27	3,456	0.9821	0.9040	0.9836	0.9982/0.9840/0.7299	0.0014/0.0162/0.0148
Visible-cell independent subset	9	1,140	0.9798	0.9344	0.9808	0.9983/0.9852/0.8197	0.0009/0.0130/0.0122

### 3.7 Processing efficiency

Computational efficiency was evaluated on the 44-image complete-tray evaluation set using the CPU environment described in Section 2.4.3. Wall-clock execution of the full-tray seed-detection stage required 241.80 s in total (5.50 s image−1), covering sliding-window inference and the point-fusion/global-NMS operations contained within the inference pipeline. Adaptive grid assignment from the fused seed-centre predictions required an additional 8.92 s (0.20 s image−1). The combined computational processing time was therefore 250.72 s for 44 images, or 5.70 s image−1. This value represents computational processing time rather than end-to-end operational time; manual four-corner calibration and human ground-truth/visibility annotation were not included.

### 3.8 Error-source analysis

Most errors were concentrated at the single–multi boundary, whereas errors involving empty cells were relatively uncommon. Among the 122 erroneous cells in the complete-tray evaluation set, 95 single-seeded cells were classified as multi-seeded, 18 multi-seeded cells were classified as single-seeded, 7 single-seeded cells were classified as empty, and 2 empty cells were classified as single-seeded. A similar error distribution was observed in the visible-cell evaluation set, where 66 of 83 errors were single-to-multi confusions and 11 were multi-to-single confusions. The consistent pattern across evaluation subsets indicates that the dominant error source was seed-number discrimination rather than empty-cell recognition.

Single-to-multi errors were mainly associated with residual duplicate responses or false detections that resulted in excessive seed-centre assignments within a single cell. Conversely, multi-to-single errors were related to missed seed centres, insufficient separation between adjacent seeds, or limited local visibility. The low frequency of empty-related errors suggests that the proposed framework was generally effective in distinguishing background regions, while further improvement is mainly required for separating closely located seeds within occupied cells.

## 4 Discussion

### 4.1 From local seed detection to full-tray quality indicators

The controlled patch-level comparison showed similar overall performance for HeatmapNet and MSRA-HeatmapNet, indicating that architecture alone did not explain the development-stage difference. More importantly, local seed-centre detection is only the first stage of full-tray sowing-quality assessment: overlapping windows can duplicate responses, while pose and perspective alter the correspondence between image coordinates and physical tray cells. Reliable cell states therefore require explicit control of both duplicate centres and seed-to-cell geometry.

The contribution of the proposed framework lies in establishing a complete transition from local seed-centre localisation to interpretable tray-level quality indicators. By integrating heatmap-based localisation, global duplicate suppression, and adaptive grid assignment, the framework converts individual seed responses into cell-level sowing states and aggregate sowing-quality metrics suitable for greenhouse production monitoring.

### 4.2 Roles of global duplicate suppression and adaptive grid assignment in full-tray analysis

Maize seeds occupy only a small fraction of high-resolution full-tray images, making accurate localisation challenging under limited pixel representation. Small-object detection remains difficult because targets contain insufficient visual information after image resizing or feature extraction (Bai et al., 2018). Patch-based slicing strategies have therefore been introduced to preserve local details and improve small-target representation in high-resolution images (Tong et al., 2020). However, overlapping patches may generate duplicate responses for the same physical target, requiring effective duplicate suppression before counting.

After duplicate removal, accurate seed-to-cell correspondence remains essential. Perspective distortion and tray pose variation introduce spatial deviations between image coordinates and physical cell locations. Geometric transformations based on homography estimation are widely used to correct such planar distortions and establish consistent coordinate mappings (Ferris et al., 2024). In addition, image-based localisation methods have demonstrated the importance of reliable spatial correspondence between visual observations and physical positions (Abanay et al., 2025).

### 4.3 Robustness and generalisation under non-ideal imaging conditions

Real greenhouse images often contain incomplete tray boundaries, view-angle deviations, partial coverage, and complex backgrounds. Such variations are common challenges in agricultural vision systems because environmental conditions and image acquisition settings can strongly influence model performance (Wang et al., 2019; Ghazal et al., 2024). Therefore, evaluation under complete and regular trays alone may not fully reflect practical acquisition conditions.

The visible-cell analysis showed that cell-state classification remained consistent within manually assessable regions of partially visible tray images. However, the retained quantitative subset was predominantly assessable: 25 of 30 images had all 128 cells available for evaluation, and the remaining five retained 120–124 assessable cells. Thus, this experiment primarily characterizes performance under limited edge loss, viewpoint variation, and non-ideal tray geometry rather than severe partial visibility.

Images with unreliable grid calibration or unclear tray boundaries were excluded from quantitative evaluation because reliable cell-level correspondence and ground truth could not be established. After excluding samples with training-source overlap, the source-image-independent subsets showed performance patterns consistent with the larger evaluation sets, but the 9-image visible-cell subset was likewise predominantly assessable (93.75%–100% per image). The visible-cell analysis therefore addresses performance within manually assessable regions under incomplete observation, whereas the source-image-independent analysis addresses direct source-image overlap. Neither constitutes validation under severe boundary loss or a new acquisition domain.

### 4.4 Methodological limitations and future directions

Several aspects require further investigation. First, multi-seeded cells remained the least stable class, with an F1-score of 0.7631 and a tray-clustered 95% confidence interval of 0.6950–0.8211. This wider uncertainty relative to the empty and single-seeded classes is consistent with the smaller number of multi-seeded observations (200 of 5,632 evaluated cells), although class imbalance alone does not explain the observed errors. The error analysis further showed contributions from missed seed centres, insufficient separation between closely positioned seeds, and residual duplicate responses. Increasing the diversity and frequency of multi-seeded cases, particularly closely spaced and visually ambiguous examples, may improve the reliability of this category.

Second, the current implementation assumes manually calibrated tray geometry and manually determined assessable cells in the visible-cell evaluation. Automatic tray/corner localisation and automatic cell-assessability determination were not evaluated and remain necessary steps toward end-to-end automation. Calibration repeatability was not independently quantified; the present workflow used one operator for calibration and a second person for quality-control review.

Third, the source-image-independent analyses were designed to reduce the influence of direct source-image overlap and should not be interpreted as external validation. All images were acquired during a single production campaign at one production site using one imaging device and one tray specification. Although the dataset contains within-domain variation in illumination, substrate appearance, tray pose, perspective distortion, and boundary visibility, these variations do not represent independent acquisition domains. Validation across different production sites or greenhouses, imaging devices, tray specifications, acquisition protocols, seasons, and maize varieties is required before broader external generalisation or deployment can be claimed.

## 5 Conclusions

This study developed a semi-automated framework integrating heatmap-based seed-centre localisation, global duplicate suppression, and four-corner-calibrated adaptive grid assignment for maize tray sowing-quality assessment. On the source-image-independent subsets, accuracy and macro F1 were 0.9821 and 0.9040 for 27 complete-tray images (3,456 cells), and 0.9798 and 0.9344 for 9 visible-cell images (1,140 assessable cells). Separately, the larger evaluation sets yielded 0.9783/0.9145 for 44 complete trays and 0.9782/0.9368 for 30 visible-cell images. Clustered and paired tray-level bootstrap analyses supported the stability of the reported cell-level results and the contributions of global NMS and adaptive grid assignment. The source-image-independent subsets remove direct source overlap but remain within the same acquisition domain and therefore do not constitute external validation (Ovadia et al., 2019). Broader validation across production sites, devices, tray specifications, acquisition conditions, seasons, and maize varieties is required before external generalisation or wider deployment can be claimed.

## Data Availability

The original contributions presented in the study are included in the article/**Supplementary Material**. Further inquiries can be directed to the corresponding author.
