# Supplementary material for: From seed centres to tray cells: a geometry-aware machine vision framework for full-tray sowing quality assessment in maize plug trays
Source: Front Plant Sci. 2026 Oct 23;17:1981493. doi: 10.3389/fpls.2026.1981493 (PMC13739767; doi:10.3389/fpls.2026.1981493)
Supplement: Supplementary file 1 [file Supplementaryfile1.docx]

Supplementary Material

*Supporting dataset composition, evaluation results, uncertainty analyses, and reproducibility information*

**Scope.** This supplementary file provides additional dataset composition, evaluation, uncertainty, ablation, efficiency, and implementation details supporting the analyses reported in the main article. It is intended to facilitate interpretation and reproducibility of the reported results. Original image pixels and the trained model checkpoint are not included in this supplementary file.

# S1. Dataset construction and annotation

**Patch-level dataset.** The final manually verified patch dataset contained 1,523 patch images and 2,681 annotated seed centres. The retained development split was used for model optimisation, checkpoint selection, response-threshold selection, and patch-level evaluation. The original patch split was not treated as source-image independent because subsequent auditing showed that patches derived from some parent images occurred in more than one development subset. A separate source-image-grouped partition was therefore used for the controlled architecture comparison.

**Supplementary Table S1. Composition of the final patch-level seed-centre dataset.**

| **Subset** | **Images** | **Seed-containing images** | **Blank images** | **Complex negative samples** | **Seed-centre points** |
| --- | --- | --- | --- | --- | --- |
| Training | 1,156 | 750 | 133 | 273 | 2,039 |
| Validation | 182 | 112 | 22 | 48 | 317 |
| Testing | 185 | 113 | 23 | 49 | 325 |
| Total | 1,523 | 975 | 178 | 370 | 2,681 |

**Full-tray evaluation data.** The full-tray evaluation design separated the main complete-tray evaluation, the visible-cell robustness evaluation, and stricter source-image-independent subsets. The complete-tray main set included all 128 cells per tray. The visible-cell evaluation included only cells manually judged to be visible and assessable. The stricter subsets excluded evaluation samples whose source identifiers overlapped with source images used for patch-level training.

**Supplementary Table S2. Full-tray cell-level evaluation datasets.**

| **Evaluation set** | **Images** | **Valid cells** | **Evaluation protocol** | **Purpose** |
| --- | --- | --- | --- | --- |
| Complete-tray main set | 44 | 5,632 | All 128 cells per tray | Main evaluation |
| Visible-cell evaluation set | 30 | 3,808 | Cells with visible = 1 | Robustness within assessable regions |
| Complete-tray independent subset | 27 | 3,456 | Overlapping source IDs excluded | Source-overlap control |
| Visible-cell independent subset | 9 | 1,140 | Overlapping source IDs excluded | Source-overlap control |

# S2. Controlled patch-level architecture comparison

HeatmapNet and MSRA-HeatmapNet were compared using the same source-image-grouped dataset partition, identical optimisation and augmentation settings, the same random seed and evaluation protocol, and independent threshold selection on the same validation set. The comparison was used only to isolate architecture-related effects; the controlled models did not replace the MSRA-HeatmapNet checkpoint used in the downstream full-tray experiments.

**Supplementary Table S3. Controlled architecture comparison on the common source-image-grouped patch test set.**

| **Model** | **Selected threshold** | **TP/FP/FN** | **Precision** | **Recall** | **F1-score** | **Mean localisation error (px)** | **Mean absolute count error** |
| --- | --- | --- | --- | --- | --- | --- | --- |
| HeatmapNet | 0.45 | 366/83/37 | 0.8151 | 0.9082 | 0.8592 | 7.451 | 0.3262 |
| MSRA-HeatmapNet | 0.80 | 358/78/45 | 0.8211 | 0.8883 | 0.8534 | 7.518 | 0.2876 |

# S3. Cell-level sowing-quality evaluation

**Complete-tray evaluation.** Across 44 complete or near-complete tray images (5,632 cells), the framework achieved an overall accuracy of 0.9783 and a macro F1-score of 0.9145. The class-specific F1-scores were 0.9975 for empty cells, 0.9830 for single-seeded cells, and 0.7631 for multi-seeded cells. The tray-level mean absolute errors of the empty, qualified (single-seeded), and multi-seeded rates were 0.0016, 0.0174, and 0.0158, respectively.

**Visible-cell evaluation.** The visible-cell robustness set contained 30 images and 3,808 assessable cells. Performance was evaluated only within manually assessable regions; images with unreliable grid calibration or unclear boundaries were excluded from quantitative evaluation rather than forced into the analysis.

**Supplementary Table S4. Cell-level performance on the visible-cell evaluation set.**

| **Evaluation set** | **Images** | **Valid cells** | **Overall accuracy** | **Macro F1** | **Weighted F1** | **F1-empty** | **F1-single** | **F1-multi** |
| --- | --- | --- | --- | --- | --- | --- | --- | --- |
| Visible-cell robustness set | 30 | 3,808 | 0.9782 | 0.9368 | 0.9793 | 0.9967 | 0.9845 | 0.8293 |

# S4. Statistical uncertainty and paired comparisons

Because multiple cell-level observations originated from the same tray image, uncertainty for the complete-tray evaluation was quantified using a tray-level clustered bootstrap. For each bootstrap replicate, 44 tray images were sampled with replacement and all cells from each selected tray were retained together. The 2.5th and 97.5th percentiles of 20,000 bootstrap replicates were used to construct 95% confidence intervals. Paired tray-level bootstrap resampling was used for downstream ablation comparisons because the same trays and cells were evaluated under both configurations.

**Supplementary Table S5. Tray-clustered bootstrap uncertainty for the complete-tray evaluation set.**

| **Metric** | **Estimate** | **95% confidence interval** |
| --- | --- | --- |
| Overall accuracy | 0.9783 | 0.9721–0.9842 |
| Macro F1 | 0.9145 | 0.8912–0.9347 |
| F1-empty | 0.9975 | 0.9950–0.9992 |
| F1-single | 0.9830 | 0.9790–0.9870 |
| F1-multi | 0.7631 | 0.6950–0.8211 |

**Supplementary Table S6. Paired tray-level bootstrap effects of downstream modules.**

| **Comparison** | **Metric** | **Difference** | **95% confidence interval** |
| --- | --- | --- | --- |
| With NMS − without NMS | Accuracy | +0.4123 | 0.3587–0.4645 |
| With NMS − without NMS | Macro F1 | +0.3711 | 0.3514–0.3901 |
| Adaptive grid − fixed grid | Accuracy | +0.2422 | 0.2195–0.2638 |
| Adaptive grid − fixed grid | Macro F1 | +0.3222 | 0.2955–0.3506 |

# S5. Ablation results

**Global duplicate suppression.** Removing global NMS substantially increased cross-window duplicate responses and degraded cell-level classification. Reintroducing global NMS reduced the candidate count from 8,089 to 4,226 and restored the complete-tray accuracy and macro F1-score to 0.9783 and 0.9145.

**Supplementary Table S7. Ablation of global duplicate suppression.**

| **Setting** | **Candidate points** | **Overall accuracy** | **Macro F1** | **Weighted F1** | **F1-empty** | **F1-single** | **F1-multi** | **Error cells** |
| --- | --- | --- | --- | --- | --- | --- | --- | --- |
| Without global NMS | 8,089 | 0.5661 | 0.5435 | 0.6427 | 0.9975 | 0.4949 | 0.1380 | 2,444 |
| With global NMS | 4,226 | 0.9783 | 0.9145 | 0.9798 | 0.9975 | 0.9830 | 0.7631 | 122 |

**Adaptive grid assignment.** The adaptive grid was compared with a fixed rectangular grid using the same retained seed centres after global NMS. The adaptive grid reduced seed-to-cell assignment errors under tray pose and perspective variation and substantially reduced tray-level rate errors.

**Supplementary Table S8. Ablation of adaptive grid assignment.**

| **Setting** | **Overall accuracy** | **Macro F1** | **F1-single** | **F1-multi** | **MAE-qualified rate** | **MAE-multi rate** | **Error cells** |
| --- | --- | --- | --- | --- | --- | --- | --- |
| Fixed rectangular grid | 0.7362 | 0.5924 | 0.7789 | 0.2392 | 0.1355 | 0.0618 | 1,486 |
| Adaptive grid | 0.9783 | 0.9145 | 0.9830 | 0.7631 | 0.0174 | 0.0158 | 122 |

# S6. Source-image-independent validation

The stricter source-image-independent subsets were constructed by excluding evaluation samples whose source identifiers overlapped with source images used for patch-level training. These analyses address direct source overlap but remain within the same acquisition domain and therefore should not be interpreted as external validation across production sites, imaging devices, tray specifications, seasons, or maize varieties.

**Supplementary Table S9. Evaluation on strict source-image-independent subsets.**

| **Evaluation set** | **Images** | **Valid cells** | **Overall accuracy** | **Macro F1** | **Weighted F1** | **Class-wise F1 (empty/single/multi)** | **Rate MAE (empty/qualified/multi)** |
| --- | --- | --- | --- | --- | --- | --- | --- |
| Complete-tray independent subset | 27 | 3,456 | 0.9821 | 0.9040 | 0.9836 | 0.9982 / 0.9840 / 0.7299 | 0.0014 / 0.0162 / 0.0148 |
| Visible-cell independent subset | 9 | 1,140 | 0.9798 | 0.9344 | 0.9808 | 0.9983 / 0.9852 / 0.8197 | 0.0009 / 0.0130 / 0.0122 |

# S7. Processing efficiency

Computational efficiency was evaluated on the 44-image complete-tray evaluation set. Full-tray seed detection, including sliding-window inference and point-fusion/global-NMS operations, required 241.80 s in total (5.50 s per image). Adaptive grid assignment from fused seed-centre predictions required an additional 8.92 s (0.20 s per image). The combined computational processing time was therefore 250.72 s for 44 images, or 5.70 s per image. Manual four-corner calibration and human ground-truth/visibility annotation were not included in this timing.

**Supplementary Table S10. Processing-efficiency summary.**

| **Stage** | **Total time (s)** | **Mean time per image (s)** | **Images** |
| --- | --- | --- | --- |
| Full-tray seed detection + point fusion/global NMS | 241.80 | 5.50 | 44 |
| Adaptive grid assignment | 8.92 | 0.20 | 44 |
| Combined computational processing | 250.72 | 5.70 | 44 |

# S8. Key implementation parameters

The following parameters are provided to clarify the configuration used for the reported experiments. The response threshold was determined from validation data, and the grid-assignment tolerance of 0.50 grid-cell units was used in the reported implementation.

**Supplementary Table S11. Key model, inference, and grid-assignment parameters.**

| **Parameter** | **Setting** |
| --- | --- |
| Original patch size | 512 × 512 pixels |
| Network input size | 256 × 256 pixels |
| Output heatmap size | 128 × 128 pixels |
| Gaussian kernel standard deviation | 2.5 |
| Optimiser | AdamW |
| Initial learning rate | 0.001 |
| Weight decay | 0.0001 |
| Learning-rate scheduler | ReduceLROnPlateau |
| Batch size | 4 |
| Loss function | Weighted MSE + BCE heatmap loss |
| Loss weights | MSE = 1.0; BCE = 0.2; BCE positive weight = 18.0 |
| Response threshold | 0.85 |
| Minimum peak distance | 6 heatmap pixels |
| Sliding-window size | 512 × 512 pixels |
| Sliding-window stride | 384 pixels |
| Window overlap | 128 pixels (25%) |
| Internal edge margin | 48 pixels |
| Global NMS distance | 26 pixels |
| Grid-assignment tolerance | 0.50 grid-cell units |

# S9. Interpretation and reproducibility notes

**Cell-state definition:** Each tray cell was classified as empty, single-seeded, or multi-seeded according to the number of de-duplicated seed centres assigned to that cell.

**Visible-cell analysis:** Only cells manually designated as visible and assessable were included in the visible-cell quantitative evaluation.

**Qualified rate:** The qualified rate corresponds to the single-seeded rate.

**Source-image independence:** The strict subsets remove direct overlap between evaluation samples and source images used for patch-level training, but they do not constitute external-domain validation.

**Ablation comparisons:** Paired tray-level bootstrap resampling was used because the same tray images and cells were evaluated under both module configurations.

**Scope of supplementary file:** This file provides tabulated supporting information. Original image pixels and the trained model checkpoint are not included.

***End of Supplementary Material***
